# Novel Variant in *ANO5* Muscular Dystrophy: Identification by Whole Genome Sequencing and Quad Analysis

**DOI:** 10.3390/genes15101300

**Published:** 2024-10-06

**Authors:** Mario Ćuk, Busra Unal, Luka Lovrenčić, McKenzie Walker, Connor P. Hayes, Feruza Abraamyan, Maja Prutki, Goran Krakar, Lidija Srkoč-Majčica, Arezou A. Ghazani

**Affiliations:** 1 Department of Pediatrics, University Hospital Centre Zagreb, 10000 Zagreb, Croatia; 2 School of Medicine, University of Zagreb, 10000 Zagreb, Croatia; 3 Division of Genetics, Brigham and Women’s Hospital, Boston, MA 02115, USA; 4 Department of Radiology, University Hospital Centre Zagreb, 10000 Zagreb, Croatia; 5 Pediatric Clinic Sabol, 10000 Zagreb, Croatia; 6 Zabok General Hospital and Croatian Veterans Hospital, 49210 Bračak, Croatia; 7 Department of Medicine, Brigham and Women’s Hospital, Boston, MA 02115, USA; 8 Harvard Medical School, Boston, MA 02115, USA

**Keywords:** *ANO5* muscle disease, preventive medicine, joint whole genome analysis

## Abstract

Background: The phenotypic spectrum of *ANO5* muscle disease ranges widely from elevated creatine kinase (CK) levels in the serum of asymptomatic individuals to progressive muscular dystrophy. Due to overlapping clinical features among muscular dystrophies, the diagnosis of *ANO5* muscle disease is established by molecular genetic tests. Early diagnosis is crucial for the clinical management of symptoms and to mitigate cardiac and musculoskeletal complications. Methods: Quad-joint analysis was performed on whole genome sequencing (WGS) data obtained from an 18-year-old female with mild myalgia and elevated CK and her unaffected parents and sister. The phenotype-driven analysis was performed to prioritize genomic alterations related to the phenotype. The zygosity-based analysis investigated compound heterozygous and *de novo* status for all variants. Results: The quad-joint WGS analysis revealed a novel pathogenic heterozygous variant, *ANO5*:c.1770_1773del (p.Phe593Metfs*15), that was paternally inherited. A second and known pathogenic heterozygous variant, *ANO5*:c.148C>T (p.Arg50*), was also present that was maternally inherited. The genome finding led to the diagnosis of autosomal recessive *ANO5* muscle disease and an early personalized clinical management for the patient regarding her cardiac and musculoskeletal health. Conclusions: This is the first report of the *ANO5*:c.1770_1773del variant in the literature. This report highlights the spectrum of *ANO5* muscle disease and describes the role of quad-joint WGS in the early diagnosis and preventive clinical management of *ANO5* muscle disease.

## 1. Introduction

*ANO5* muscle disease is one of the most common limb–girdle muscular dystrophies with a prevalence of 0.27/100,000 to 2:100,000 [1,2]. The clinical presentations of *ANO5* muscle disease exhibit a wide spectrum of phenotypes, including elevated CK in asymptomatic individuals, upper and lower limb weakness, and muscular atrophy [1,3,4]. The *ANO5* gene (MIM ID: *608662) on chromosome 11p14.3 belongs to the anoctamin family of calcium-activated chloride channels and causes two muscle diseases. Autosomal recessive limb–girdle muscular dystrophy type 2L (LGMD2L) (MIM ID: 611307) is characterized by late-onset (range 15–70 years) proximal lower-limb weakness [4,5]. Miyoshi muscular dystrophy type 3 (MMD3) (MIM ID: 613319) is characterized by early-adult-onset calf distal myopathy (around 20 years of age) [1,2,6]. About 10–30% of the patients with *ANO5* muscle disease exhibit cardiac involvement varying from subclinical arrhythmia to symptomatic cardiomyopathy [7].

Early diagnosis is crucial for developing a personalized clinical management plan for patients with *ANO5* muscle disease. Neuromuscular and cardiac assessment at the initial diagnosis is essential to set a baseline for monitoring the symptom progress. Heavy muscle training and the use of statins should be avoided to prevent further muscle damage [8]. An *ANO5* muscle disease diagnosis is established by identifying biallelic *ANO5* pathogenic variants. 

Here, we describe an 18-year-old female with mild symptoms diagnosed with *ANO5* muscle disease through quad whole genome analysis (quad WGS). The molecular analysis identified a novel pathogenic variant in *ANO5* that has not been previously reported in the literature. This study also describes the genome analysis strategy and highlights the crucial role of early molecular diagnosis in guiding the clinical management of *ANO5* muscle diseases. 

## 2. Materials and Methods

### 2.1. Participants

The proband, her unaffected parents, and her unaffected sister were enrolled in the CROseq genome program. The CROseq genome program is a research program between Brigham and Women’s Hospital (BWH) (Boston, MA, USA) and the Department of Pediatrics, University Hospital Centre Zagreb (Zagreb, Croatia), funded by the Mila za Sve Foundation (Rijeka, Croatia). Consent protocols were developed specifically for the CROseq research program and included broad participants (the proband and family members) and broad genomic findings (all actionable findings related to phenotype and secondary genes ACMG SF v.3.0). Informed consent was obtained at the University Hospital Center Zagreb per the protocol approved by the Institutional Review Board.

### 2.2. Whole Genome Sequencing

WGS was performed as previously described [9,10]. Briefly, DNA from 2 mL of peripheral blood was isolated at the purity ratio of 1.75–2.0. Following the robotic library preparation, sequencing was performed on the Illumina NovaSeq 6000 platform (San Diego, CA, USA) with a 40X average coverage depth.

### 2.3. Joint Quad WGS Analysis

The genomic sequence data obtained from the proband, unaffected sister, and unaffected parents were analyzed at BWH. Quad WGS joint assessment in conjugation with the phenotype-based assessment was carried out. Variant prioritization was performed according to the genotype–phenotype association. The proband’s clinical features informed the analysis using the following Human Phenotype Ontology (HPO) terms: elevated circulating creatine kinase concentration (HP:0003236), exercise-induced muscle cramps (HP:0003710), muscle spasm (HP:0003394), chronic fatigue (HP:0012432), hypersomnia (HP:0100786), pelvic girdle muscle atrophy (HP:0008988), quadriceps muscle weakness (HP:0003731).

All genomic regions were investigated in the analysis. A quality assessment was performed to exclude low-quality and failed variants. This technical assessment was performed manually after variant calling based on variant allelic fraction and sequencing depth. Allele frequencies for each allele were assessed using the gnomAD (v2.1.1) genome database. Missense variants were assessed using *in silico* aggregate prediction scores from CADD, REVEL, Polyphen, SIFT, MutationTaster, Mutation Assessor, FATHMM, FITCONS, GENOCANYON, dbscSNV ADA, and dbscSNV RF. Splice variants were assessed with the Splice AI prediction algorithm.

“A *de novo* variant analysis” was conducted to identify variants associated with the patient‘s phenotype present in the proband but not in the unaffected parents and the sister. A separate zygosity-focused analysis was carried out to investigate compound heterozygous and homozygous variants. In this analysis, variants in genes with autosomal recessive inheritance were identified. Candidate variants associated with the patient‘s phenotypes were prioritized. 

Candidate variants were classified according to ACMG-AMP guidelines [11]. Variant frequencies were assessed by using the reported frequencies in the gnomAD database (v2.1.1) to assess rarity. Genes were deemed loss-of-function intolerant if pLI = 1 and/or o/e < 0.35 from gnomAD. An aggregated in silico prediction score higher than 0.7 indicates a deleterious effect, and scores less than 0.15 are considered the sign of a benign effect. 

## 3. Results

### 3.1. Clinical Presentation

**Figure 1:**
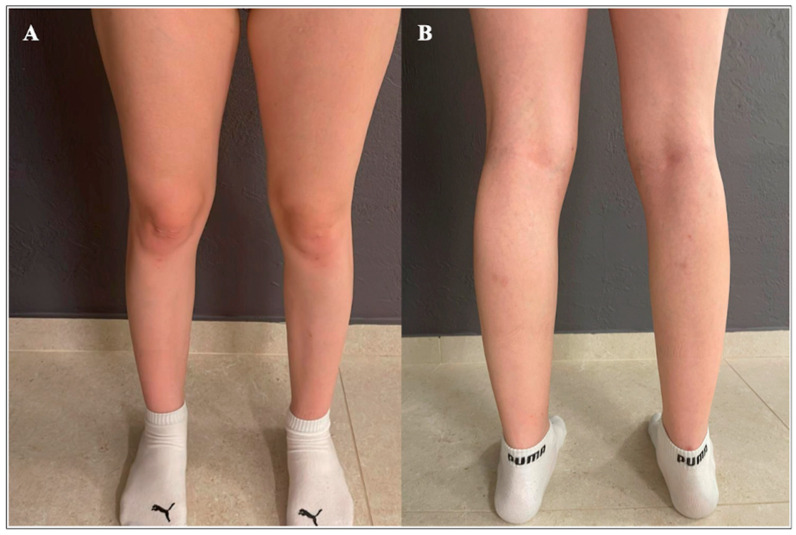
Lower extremities at the age of 18 years. (**A**) From the front: clinical findings were unremarkable, except for potential mild hypotrophy of the lower leg muscles. (**B**) From the back: clinical findings were unremarkable, with no calf hypertrophy.

An 18-year-old female presented to the Department of Pediatrics in University Hospital Centre Zagreb with exercise-induced myalgia, cramps, chronic fatigue, and frequent infections. Parents were nonconsanguineous and of south European origin. She was the only family member with such symptoms. She had one sister of 22 years of age who was unaffected. In the proband, muscular symptoms started at 17 years of age with muscle pain and cramps in the legs after prolonged walking. Laboratory workup revealed both elevated CK (maximum CK 2747 IU/L; normal < 249) and myoglobin levels (maximum MYO-S 132 μg/L; normal < 90). At the time of the last examination, at the age of 18 years, she was ambulatory and had no muscle weakness. She exhibited mild hypotrophy of the lower leg muscles (Figure 1). Magnetic resonance imaging (MRI) of the upper arm, thigh, and calf skeletal muscles was unremarkable. She did not have any clinical signs of myocardium involvement and CM-MB, and both EKG and heart ultrasound were normal.

### 3.2. The Quad WGS Joint Analysis

Quad WGS analysis was performed on the genomic sequencing data obtained from the proband, her unaffected parents, and her unaffected sister. A total of 37,550 variants in 14,315 genes were identified in HPO-based analysis in the proband. 

**Figure 2:**
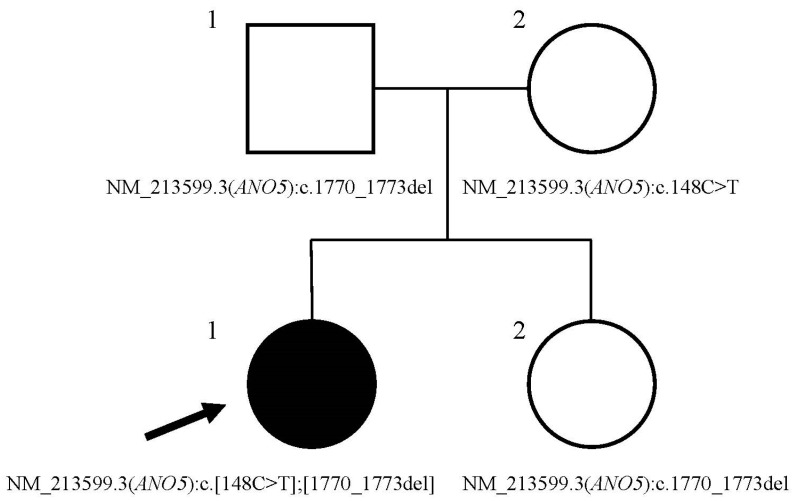
The pedigree of the family and the status of *ANO5* variants. The pedigree shows the index patient with *ANO5* muscle disease and *ANO5* genotypes in the family. Het stands for heterozygous state. Circles and squares represent female and male individuals, respectively. The arrow indicates the index patient. The black filling color denotes *ANO5* muscle disease. The *ANO5* genotype for each individual is reported under circles or squares.

The compound heterozygosity analysis detected two recessive pathogenic variants in the *ANO5* gene. One variant was NM_213599.3(*ANO5*):c.1770_1773del (p.Phe593Metfs*15), located on exon 16 of the gene. This four-bp deletion causes a frameshift that leads to a premature stop codon (Supplementary Figures S1A and S2A). Loss of function in *ANO5* is a well-known mechanism of *ANO5* muscular disease. This variant was paternally inherited by the proband and her sister (Figure 2). This is a novel variant; it has not been reported in gnomAD, ClinVar, or PubMed. The variant was classified as likely pathogenic based on ACMG criteria (PVS1, PM2). The second variant, a maternally inherited NM_213599.3(*ANO5*):c.148C>T (p.Arg50*), is located on exon 4 of *ANO5*. This variant results in a premature stop codon and is predicted to cause nonsense-mediated decay (Supplementary Figures S1B and S2B), which leads to loss of function. The variant is absent in gnomAD and has been reported in trans with another *ANO5* variant in an individual affected with pseudo-metabolic myopathy (ClinVar Accession: SCV000645874.5). Therefore, it has been reported as pathogenic by multiple users in the ClinVar database (ACMG criteria: PVS1, PM2, PM3; Variation ID: 468825). The analysis to identify *de novo* variants did not reveal any candidate variant related to muscle disease. 

### 3.3. Clinical Management

Early diagnosis in our patient herein enables timely disease management. A low-intensity aerobic exercise regimen was suggested to improve cardiovascular fitness and muscle function with instructions to avoid strenuous anaerobic exercise. A cardiology follow-up program, regular ECG, and heart ultrasound were discussed, as myocardium can become affected at any stage of the disease. 

## 4. Discussion

*ANO5* encodes the chloride channel protein Anoctamine-5, which plays a role in the repair of the muscle cell membrane [12]. *ANO5* is expressed in skeletal muscle, myocardium, bone, and cartilage. The encoded transmembrane protein is located on the endoplasmic reticulum, which promotes the recruitment of annexin to the damaged endoplasmic reticulum, an essential step in endoplasmic reticulum repair [13,14]. Gain of function variants in the *ANO5* gene cause autosomal dominant gnathodiaphyseal dysplasia [15,16] characterized by jaw dysplasia, fragile bones, and cortical thickening of long bones [17]. Loss-of-function variants in *ANO5* cause autosomal recessive muscular diseases of Miyoshi muscular dystrophy 3 (MMD3) or limb–girdle muscular dystrophy 12 (LGMDR12).

MMD3 is a subtype of muscular dystrophy characterized by atrophy and weakness of calves and forearm muscles that results in decreased grip strength and an inability to stand on toes, with an intact ability to stand on heels [18]. MMD3 typically involves hyperCKemia and asymmetrical myopathic muscle dystrophy [19]. LGMDR12 is characterized by proximal muscular dystrophy, asymmetrical involvement of the thigh muscles which results in difficulty walking, and hyperCKemia [20]. Isolated hyperCKemia and the pseudometabolic phenotype (PMP) can progress to MMD3 or LGMDR12. The two phenotypes can also converge over time, leading to both proximal and distal muscle weakness in some patients. Age of onset is variable for the muscular phenotype, reportedly ranging from teenage years to late adulthood [4,21]. Females have a milder phenotype, with a later age of onset than males [21]. Cardiac involvement is common in *ANO5* muscular disease and involves arrhythmias, cardiomyopathy, and/or left ventricular dysfunction [4,22].

Here, we report a patient with a novel variant in the *ANO5* gene identified by WGS and quad genome analysis. This rare variant is in a compound heterozygous state in the proband with a previously well-known pathogenic variant. Our patient exhibited exercise-induced muscle pain and cramps consistent with the phenotype of *ANO5* muscular disease. She exhibited a relatively early age of onset for females. At the time of diagnosis, no signs of myocardium involvement were present, and muscle MR was unremarkable, without any signs of fibrofatty replacement. 

The previously known *ANO5*:c.148C>T variant has been documented as a compound heterozygous with other *ANO5* variants in three cases suspected of LGMD. This includes a male with distal lower limb weakness at the age of 47 (with a second reported allele of *ANO5*:c.1898+1G>A) [23]; a female with an onset of PMP at the age of 15 (with a second reported allele of *ANO5*:c.191dupA) [3], and a male with proximal lower limb weakness at the age of 20 (with a second reported allele of *ANO5*:c.191dupA) [4]. All patients were ambulatory without signs of upper limb involvement.

Known causes of muscle diseases with elevated CK and clinical presentations similar to those in our patient have a broad differential diagnosis. In our patient, using non-invasive and routine tests, some of the treatable diseases were first ruled out. They include late-onset Pompe disease (ruled out by a normal urinary glucose tetrasaccharide and dried blood spot acid α-glucosidase enzyme activity), and myoadenylate deaminase deficiency (ruled out by a measurement of plasma lactate and ammonia concentrations after forearm ischemic exercise). Before any muscle biopsy and complete work of muscle disorders, based on the institutional protocol, the patient was enrolled in the CROseq program for joint WGS analysis, which did not identify variants in any of the genes associated with distal myopathies in the differential diagnosis of *ANO5* muscle disease [2] (i.e., *DYSF*, *GNE*, *LDB3*, *MYH7*, *MYOT*, *TIA1*, *TTN*). The recommended molecular approach for establishing the diagnosis of distal myopathies, including *ANO5* muscle disease, generally includes a multigene panel or comprehensive genomic testing, with exome sequencing being more common than genome sequencing. 

In conclusion, we describe the first report of the *ANO5*:c.1770_1773del nonsense variant associated with mild muscular dystrophy. This finding broadens the variant spectrum of the *ANO5*-associated muscle disease and highlights the utility of whole genome joint analysis in the diagnosis of this muscular disorder. Early diagnosis can improve quality of life and prevent exercise that may be of high risk to a patient’s health. 

## Data Availability

The data presented in this study are available on request from the corresponding author due to BWH institutional and GDPR privacy policies.

## Supplementary Materials

The following supporting information can be downloaded at: https://www.mdpi.com/article/10.3390/genes15101300/s1, Figure S1: Variant sequencing details. (A) The genome location, exon number, and coordinates are shown for the variant NM_213599.3(*ANO5*):c.1770_1773delp.(Phe593Metfs*15) (A) and the variant NM_213599.3(*ANO5*):c.148C>T(p.Arg50*) (B). Figure S2: The protein consequences of null variants in this study. (A) shows the reference and altered proteins for NM_213599.3 (*ANO5*):c.1770_1773del p.(Phe593Metfs*15). (B) shows the reference and altered proteins for NM_213599.3 (*ANO5*):c.148C>T (p.Arg50*).
