# Supplementary material for: Novel Variant in ANO5 Muscular Dystrophy: Identification by Whole Genome Sequencing and Quad Analysis
Source: Genes (Basel). 2024 Oct 6;15(10):1300. doi: 10.3390/genes15101300 (PMC11507210; doi:10.3390/genes15101300)
Supplement: Supplementary file 1 [file genes-15-01300-s001.zip › genes-3227288-supplementary.pdf]

genes

MDPI

Supplementary

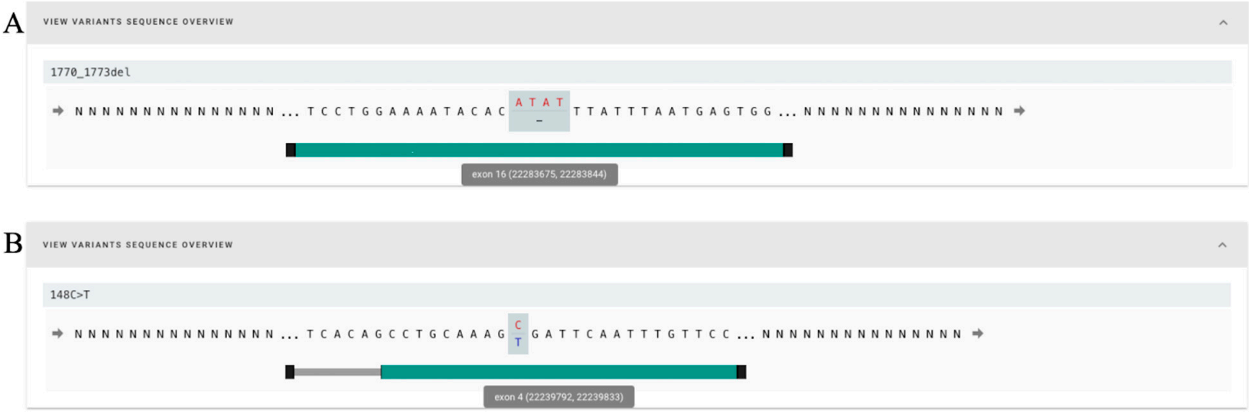

Supplementary Figure S1. Variant sequencing details. (A) The genome location, exon number, and coordinates are shown for the variant NM_213599.3 (*ANO5*):c.1770_1773del p.(Phe593Metfs*15) (A) and the variant NM_213599.3 (*ANO5*):c.148C>T (p.Arg50*) (B).

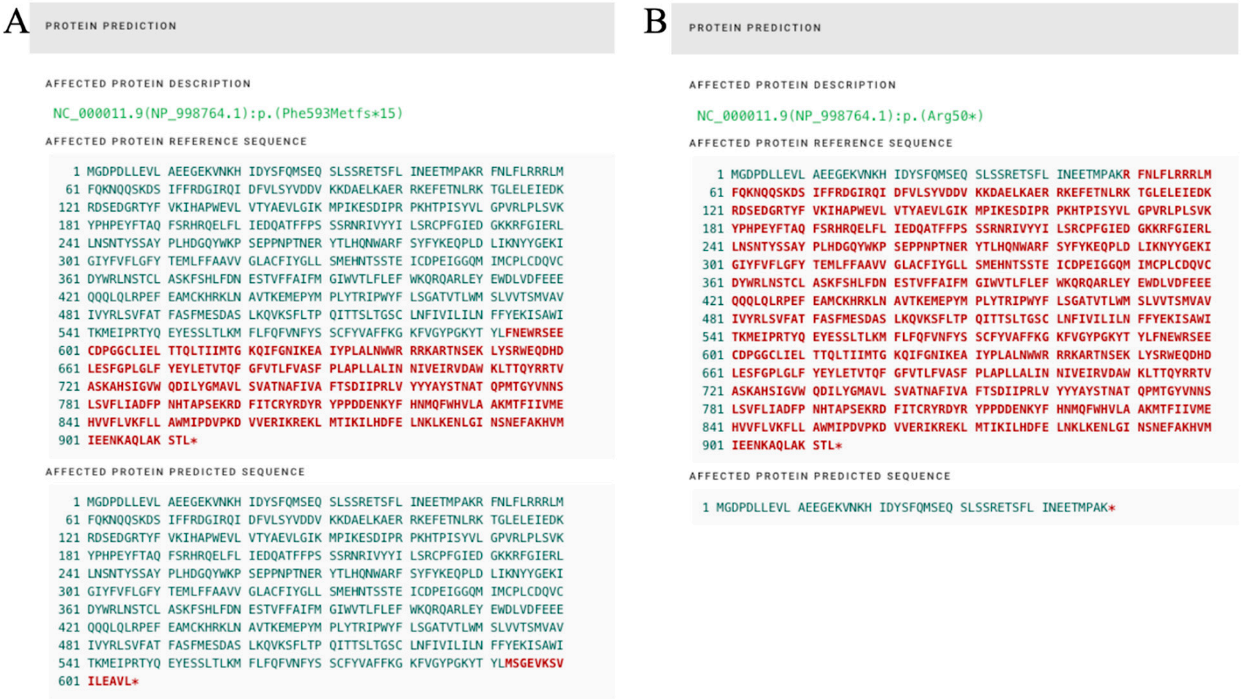

Supplementary Figure S2. The protein consequences of null variants in this study. (A) shows the reference and altered proteins for NM_213599.3 (ANO5):c.1770_1773del p.(Phe593Metfs*15). (B) shows the reference and altered proteins for NM_213599.3 (ANO5):c.148C>T (p.Arg50*).
